# Optical Anisotropy as a Probe of Proton Ordering at Ice Surfaces

**DOI:** 10.1021/acs.jctc.6c00630

**Published:** 2026-05-18

**Authors:** Alessia Muroni, Ding Pan, Marco Govoni, Ihor Kupchak, Olivia Pulci

**Affiliations:** † Department of Physics, University of Rome “Tor Vergata” and INFN, Via della Ricerca Scientifica 1, Rome 00133, Italy; ‡ Department of Physics and Department of Chemistry, 58207 Hong Kong University of Science and Technology, Clear Water Bay, Kowloon 999077, Hong Kong; § Department of Physics, Computer Science and Mathematics, 9306 University of Modena and Reggio Emilia, Via Campi 213/a, Modena 41125, Italy; ∥ V. Lashkaryov Institute of Semiconductor Physics, National Academy of Sciences of Ukraine, pr. Nauky 45, Kyiv 03680, Ukraine

## Abstract

Ice surfaces play a central role in climate processes, astrochemistry, and materials science, yet their microscopic structure remains elusive. In particular, the degree of proton ordering at ice Ih surfaces critically influences surface reactivity, stability, and phase transitions. In this work, we employ advanced computational techniques—density functional theory to optimize equilibrium geometries, and many-body perturbation theory (GW and Bethe–Salpeter equation) to describe electronic and optical properties—to investigate ordered and partially disordered thin films of hexagonal ice (Ih). First, we analyzed six surface models featuring distinct arrangements of dangling OH bonds, quantified via an order parameter, and computed their Reflectance Anisotropy spectra, which exhibit a pronounced dependence on proton ordering. Among these, two representative models, the Ih-striped and Ih-low-ordered surfaces, emerge as the most stable. For these cases, we demonstrate that proton ordering governs the anisotropy of the optical response: the striped surface supports strongly directional excitonic states, in contrast to the nearly isotropic excitons observed in the low-ordered surface. Our results establish optical anisotropy as a robust fingerprint of proton order, providing a theoretical benchmark for polarization-resolved spectroscopic studies of ice. Furthermore, we show that excitonic effects serve as a sensitive probe of surface proton configurations, paving the way for experimental discrimination between competing models of ice surfaces under cryogenic conditions.

## Graphical abstract

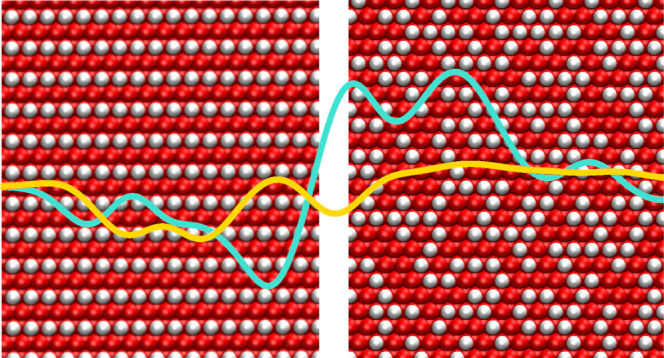

## Introduction

The study of ice and its various phases is of fundamental importance across multiple scientific disciplines, including climate and polar sciences, geophysics, astrochemistry, and solid-state physics.^1–5^ In polar science, for example, characterizing and modeling ice is crucial to understanding glacier melting processes and improving climate change predictions.^6,7^ In astrochemistry, the analysis of interstellar ices offers key insights into chemical reactions occurring in space and their role in the formation of prebiotic molecules.^8,9^

The intriguing properties of ice arise not only from its bulk structure, but also from the characteristics of its surfaces, which play a crucial role in numerous natural processes. At the atomic level, ice surfaces feature dangling OH bonds, which act as highly reactive centers, facilitating interaction with adsorbed molecules.^10,11^ Unlike the bulk phase, which is typically proton-disordered, ice surfaces may exhibit varying degrees of proton order, significantly affecting their structural and chemical behavior. Experimentally, the transition from proton-disordered ice Ih to the proton-ordered ice XI has been observed below 72 K,^12,13^ while theoretical models predict this transition near 98 K.^14,15^ Although this transition is intrinsically slow under natural conditions, it can be accelerated by dopants such as KOH.^12,13,16^ Proton ordering directly influences the arrangement of dangling OH groups at the surface, with implications for surface stability and reactivity. Theoretical models, such as Fletcher’s striped configuration,^17^ propose ordered arrangements of the ice Ih surface and offer valuable insight into atomic-scale organization under low proton disorder. However, Fletcher’s model does not represent the only possible ground-state structure, as alternative reconstructions have also been proposed.^18,19^

Accurate analysis of ice surfaces presents substantial experimental challenges.^20^ Reliable data acquisition requires techniques that are highly sensitive to the topmost surface layers while avoiding invasive or destructive methods. Moreover, such investigations must be conducted at temperatures below the surface premelting threshold, typically under 200 K.^21^ In this context, the Tammann temperature, as discussed by Buch and Tosatti,^22^ serves as a useful reference for assessing the stability limits of ice surfaces.

To explore ice surfaces and the transition between proton disorder and order, several experimental techniques have been developed. Among these, helium scattering and sum-frequency generation (SFG) spectroscopy provide detailed structural information,^23^ while infrared and Raman spectroscopy yield valuable insights into molecular dynamics.^24–26^ Nevertheless, experimental data on ice surfaces remain scarce, highlighting the role of computational studies. In this context, Berrens and co-workers applied large-scale molecular dynamics simulations based on a machine-learning potential trained on ab initio data, in order to achieve microscopic insight into the SFG spectrum and surface proton ordering.^27^ Numerous theoretical studies have investigated water and ice using methods such as the GW approximation (with G single particle Green’s function and W screened Coulomb interaction), the Bethe–Salpeter equation (BSE), and time-dependent density functional theory, enabling access to ground- and excited-state properties that are difficult to measure experimentally.^28–38^ However, studies specifically focused on the optical properties of ice surfaces remain comparatively scarce.

In this work, following,^18^ we analyzed six models of thin hexagonal ice films, each characterized by a distinct value of the order parameter C_OH_, which describes the distribution of dangling OH bonds.^18,19^ Equilibrium geometries and reflectance anisotropy spectroscopy (RAS) spectra were computed using density functional theory (DFT).^39–41^ The two most stable surface models, both with order parameter C_OH_ = 2.00, were further investigated using state-of-the-art many-body perturbation theory, including the GW approximation for quasi-particle corrections and the BSE for optical excitations.^42^ By analyzing the excitonic response of the two surface models, we demonstrate that the striped and the Ih surfaces exhibit distinct optical behaviors, with the first exciton serving as a fingerprint of the hydrogen ordering.

## Results

### Surfaces with Varying Order Parameter

Following,^18^ six different hexagonal ice (Ih) surface models were constructed, each based on a six-layer orthorhombic supercell containing 864 atoms. These models differ solely in the spatial arrangement of dangling OH bonds at the surface, which is quantified by the order parameter C_OH_ ^18,19^

1 $$C_{OH}=\frac{1}{N_{OH}}\sum_{i=1}^{N_{OH}}c_{i}$$

where *N*_OH_ is the total number of dangling OH bonds on the surface, and *c*_*i*_ denotes the number of nearest-neighbor dangling OH bonds surrounding the *i*-th dangling bond. All models maintain the same total number of OH dangling bonds (12), but differ in their distribution. The higher the *C*_OH_, the more homogeneous the distribution of OH dangling bonds. Using the supercell approach, a vacuum region of 12 Å was introduced into the unit cell in order to prevent interactions between the replicas (Figure SI 1, Supporting Information). A top-view of the DFT-optimized surface structures is shown in Figure 1, illustrating the variation in OH bond distribution across the six-layer supercells.

**Figure 1:**
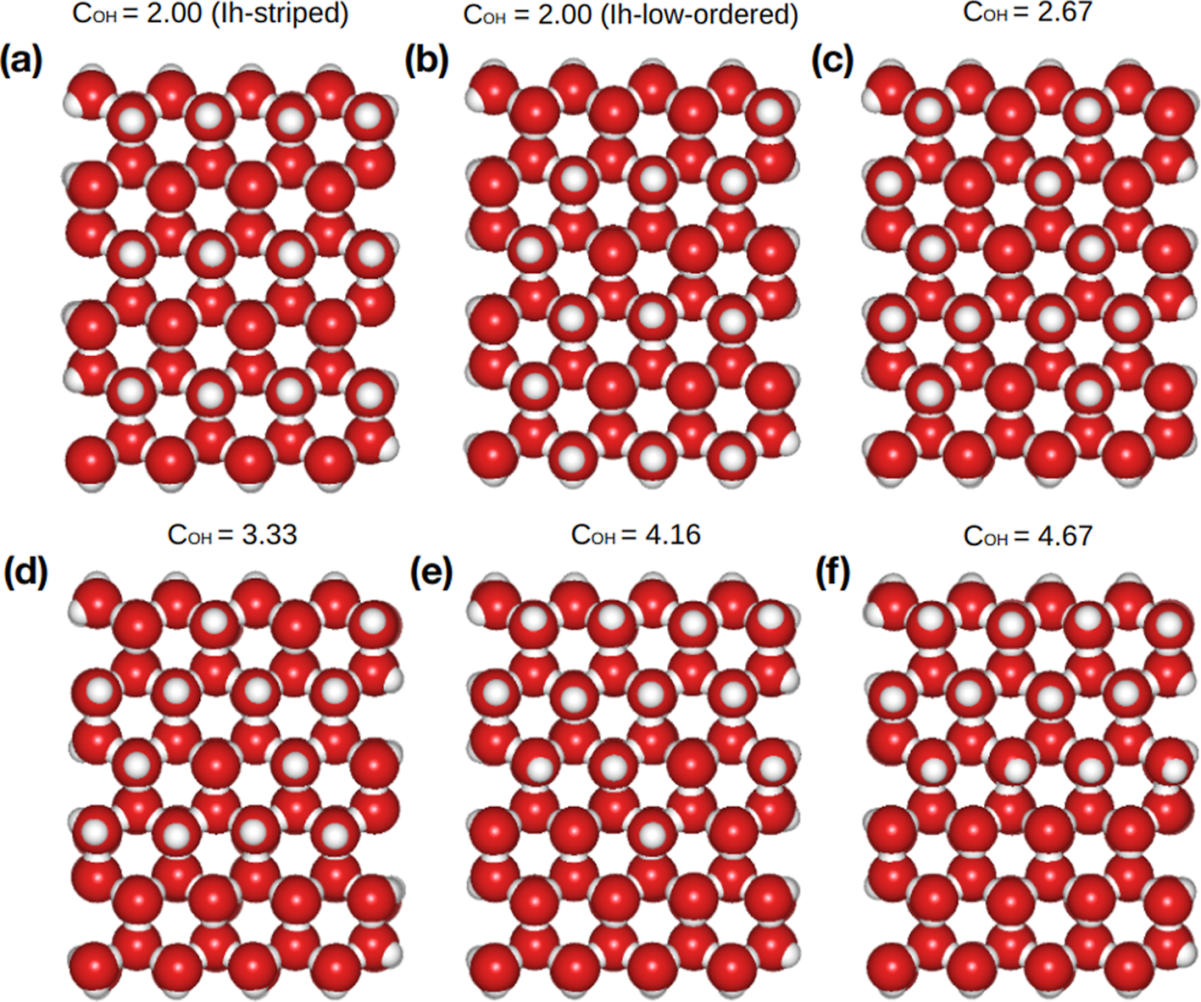
Top view of the six-layer orthorhombic supercells representing the DFT-optimized ice surfaces with different order parameters. Red and white spheres represent oxygen and hydrogen atoms, respectively.

In Figure 1a, the hydrogen atoms are arranged in a highly ordered striped pattern, consistent with the model proposed by Fletcher,^17^ with rows of dangling OH groups aligned along the surface. By contrast, the structure shown in Figure 1b, although characterized by the same C_OH_ value, does not exhibit this long-range ordering along the *x* direction and appears less regularly patterned. To distinguish between these two configurations with identical C_OH_, we refer to the structures in Figure 1a,b as Ih-striped and Ih-low-ordered, respectively.The total energies per *H*_2_ *O* molecule for the six models were computed, and their relative values with respect to the most stable configuration (the Ih-low-ordered surface) are reported in Table 1.

**Table 1:** Total Energy per H_2_O Molecule for the Six Ice Surface Models. Labels Correspond to Those in Figure 1 ^a^

surface label	C_OH_	Δ*E*_tot_/H_2_O (meV)
(a)	2.00	0.6
(b)	2.00	0.0
(c)	2.67	3.7
(d)	3.33	7.9
(e)	4.16	13.1
(f)	4.67	17.5

a The zero is taken at the total energy of the Ih-low-ordered one.

As expected, only minor differences are observed in the total energies, as the structural variations involve the hydrogen sublattice.

Although recent STM images reveal that the real ice surface is more complex than the idealized models considered here due to surface reconstruction, the experiments confirm that the order parameter *C*_OH_ correlates with the stability of the proton arrangement.^20^ Consequently, our choice of models spanning C_OH_ values from 2.00 to 4.67 enables a systematic characterization of the ice Ih surface energy landscape, ranging from the minimum-energy ordered state at C_OH_ = 2.00, through the statistically random arrangement at C_OH_ ≈3, to higher values that capture the linear increase in formation energy associated with enhanced proton clustering.^18,19^ The small energy difference between the two C_OH_ = 2.00 configurations indicates that both are thermally accessible and may coexist in the form of locally ordered domains. Consistently, STM measurements reveal heterogeneous surfaces exhibiting local proton ordering rather than a unique long-range periodic structure. In contrast, configurations with large C_OH_, which are significantly higher in energy, are unlikely to represent equilibrium surface terminations and are included here primarily to probe systematic energetic and optical trends.

The reflectance anisotropy spectroscopy (see Methods section) spectra of the six surfaces, calculated at the independent-particle level as

2 $$RAS=\frac{R_{y}-R_{x}}{R}$$

and shown in Figure 2, reveal pronounced differences in the optical response among the models, indicating that even subtle variations in surface proton ordering are reflected in the optical anisotropy, which is highly sensitive to the local hydrogen configuration.

**Figure 2:**
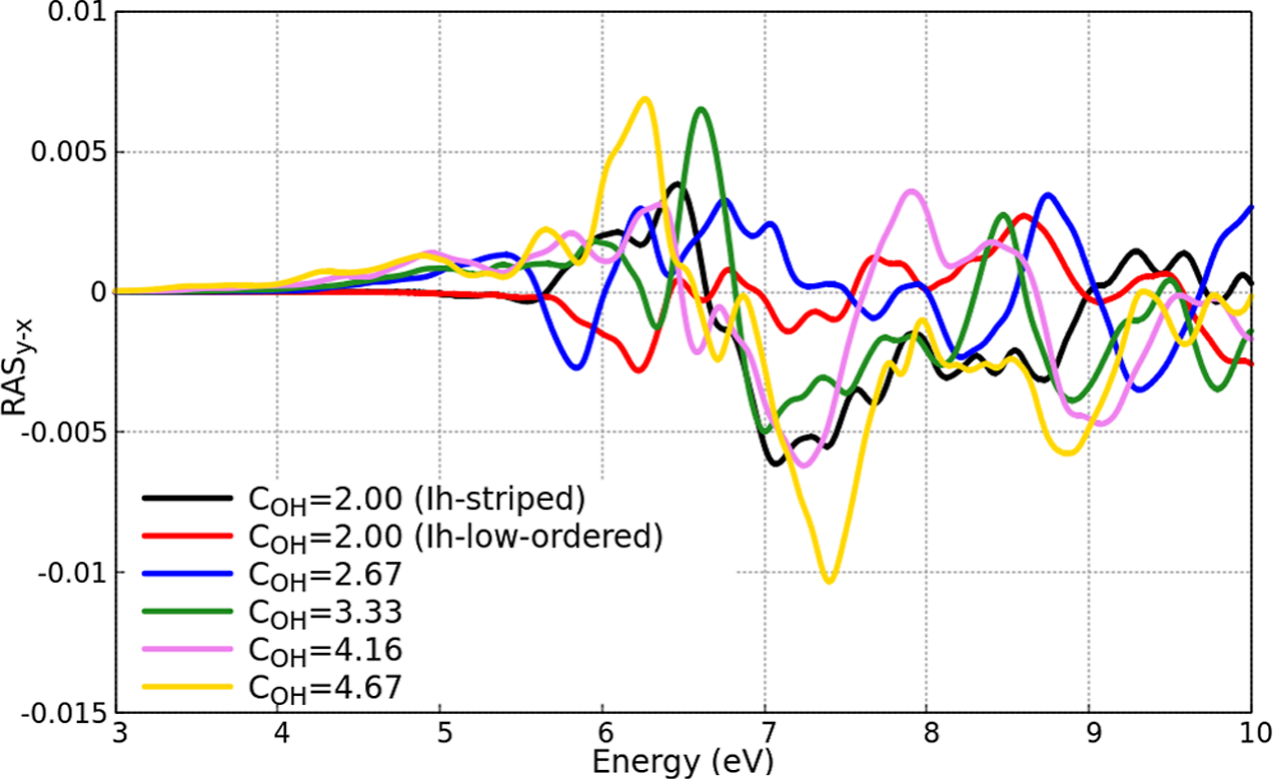
DFT RAS spectra calculated for the six ice surface models, each with a different value of the order parameter C_OH_. The x direction is aligned with the hydrogen chains in the striped model.

Although RAS spectra are inherently complex to interpret, our preliminary results demonstrate that distinct proton arrangements yield clearly distinguishable spectral features. This sensitivity to proton order highlights the potential of polarization-resolved optical spectroscopy as a diagnostic tool for probing surface reconstructions and phase transitions, such as the Ih–XI transformation relevant in cryogenic environments. Typical RAS signals for insulating surfaces are on the order of 10^–3^, as reported, for example, for diamond(001) (2 × 1)^42^ and GaAs(110) surfaces.^43^ The magnitude of the anisotropic features predicted in this work is comparable, suggesting that the optical fingerprints associated with proton ordering should be experimentally accessible under suitable conditions.

A direct comparison with future optical experiments should take into account that the proposed models represent idealized, static surface configurations, whereas real ice Ih surfaces are expected to exhibit surface reconstruction, local domain heterogeneity, and dynamical proton disorder, as observed in recent STM experiments.^20^ In realistic conditions, the optical response would therefore arise from a statistical ensemble of coexisting domains with different degrees of proton order. In this context, the calculated structures should be regarded as limiting cases, providing reference fingerprints for distinct proton-ordering motifs, whose superposition may offer insight into the local structure of heterogeneous ice surfaces.

Since DFT is limited in accurately predicting the peak positions and intensities in optical spectra, a more rigorous treatment requires many-body perturbation theory methods such as GW and BSE. However, applying these approaches to supercells containing 864 atoms is computationally prohibitive. Therefore, the post-DFT analysis was restricted to a reduced model of the two most stable surface models: Figure 1a,b.

### Many-Body Effects on the Ih-Striped and Ih-Low-Ordered Surfaces

To investigate electronic and optical properties beyond DFT, we focus on the two most representative models from the six Ih surfaces described previously: Fletcher’s striped model,^17,22^ and the low-ordered model,^18,19^ shown respectively in Figure 1a,b. They also exhibit the lowest order parameter (C_OH_ = 2.00). Corresponding reduced supercells, to make the calculations feasible, were constructed: a 4-layer hexagonal supercell containing 96 atoms for the Ih-striped surface, and a 4-layer orthorhombic supercell with 192 atoms for the Ih-low-ordered surface. These smaller models retain the essential structural features of the full systems, ensuring the reliability of the many-body calculations.

GW calculations performed on both models yield a consistent quasi-particle band gap increase, Δ*E*, of approximately 3.5 eV, resulting in a final GW gap of 8.6 eV for both surfaces. These values are comparable with previous theoretical studies on water and bulk ice.^28,32,37,44^ A more refined estimation of the band gap would require a self-consistent quasiparticle GW approach including vertex corrections, as well as the inclusion of zero-phonon renormalization,^33^ which are beyond current computational capabilities.

The in-plane optical absorbance of the two surfaces was computed at the BSE level to include excitonic effects, using the following expression

3 $$A\left(\omega \right)=\frac{\omega }{c}a_{3}\epsilon_{2}^{∥}\left(\omega \right)$$

where *c* is the speed of light, a_3_ the supercell dimension along the *z*-axis, and ϵ_2_^∥^ is the imaginary part of the in-plane dielectric function. The absorbance spectra for the Ih-striped and Ih-low-ordered surfaces, calculated by solving the Bethe–Salpeter Equation to take into account excitonic effects, are shown in Figure 3a. Although the overall spectral shapes are similar, they exhibit sizable differences in the intensity and position of the peaks. These peaks, appearing around 6.5 to 7 eV and located below the *G*_0_ *W*_0_ gap, correspond to bound excitons and can be attributed to O → H transitions involving surface-localized states near the band edge, originating from oxygen lone-pair orbitals and terminating in unoccupied hydrogen-derived states, as confirmed by the exciton analysis in the Supporting Information. Moreover, the differences between the optical responses of the two surfaces are amplified when calculating the polarization-resolved absorbance. In particular, the anisotropy of the optical response, measured as *A*_*y*_(ω) – *A*_*x*_(ω) for the in-plane absorbance provides clear fingerprints of the hydrogen ordering, as illustrated in Figures 3b and SI4c. In the Ih-striped surface, the ordered proton stripes along the *x*-direction give rise to a pronounced anisotropic response, whereas the Ih-low-ordered surface, lacking long-range proton order, exhibits a nearly isotropic behavior in both polarizability and absorbance. This demonstrates the role of proton arrangement in modulating the in-plane optical response. Polarization-resolved experiments, though challenging, could definitively help distinguish between the two surfaces.

**Figure 3:**
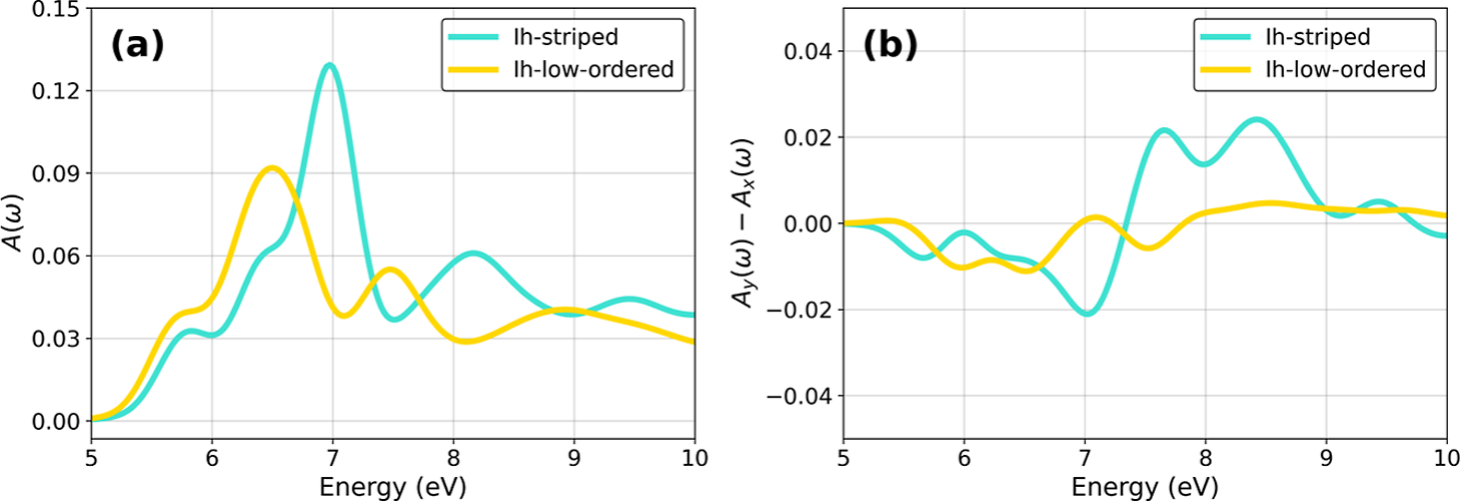
Absorbance spectra of the striped and low-ordered model, calculated at the BSE level under different polarization conditions: (a) in-plane absorbance and (b) absorbance anisotropy between *y*- and *x*-polarized light.

To gain further insight into the spatial character of these excitations, the excitonic wave functions for the two surfaces are analyzed. Figure 4 reports the excitonic wave functions associated with representative excitons for both surfaces, providing a detailed view of exciton orientation and delocalization in the two surface configurations. In particular, Figure 4a,b corresponds to the top and side views of the exciton wave function of the Ih-striped thin-film, with energy *E*_exc_ = 5.7 eV, while Figure 4c,d shows the top and side views of the exciton wave function of the Ih-low-ordered thin-film, with energy *E*_exc_ = 5.8 eV. For the Ih-striped surface, the exciton is doubly degenerate due to the hexagonal symmetry and ordered proton configuration, resulting in anisotropic delocalization along the *x*-direction. In contrast, the Ih-low-ordered surface lacks this degeneracy, consistent with its reduced long-range order and more isotropic exciton distribution. These differences in excitonic structure further highlight the impact of surface hydrogen arrangement on the electronic and optical properties.

**Figure 4:**
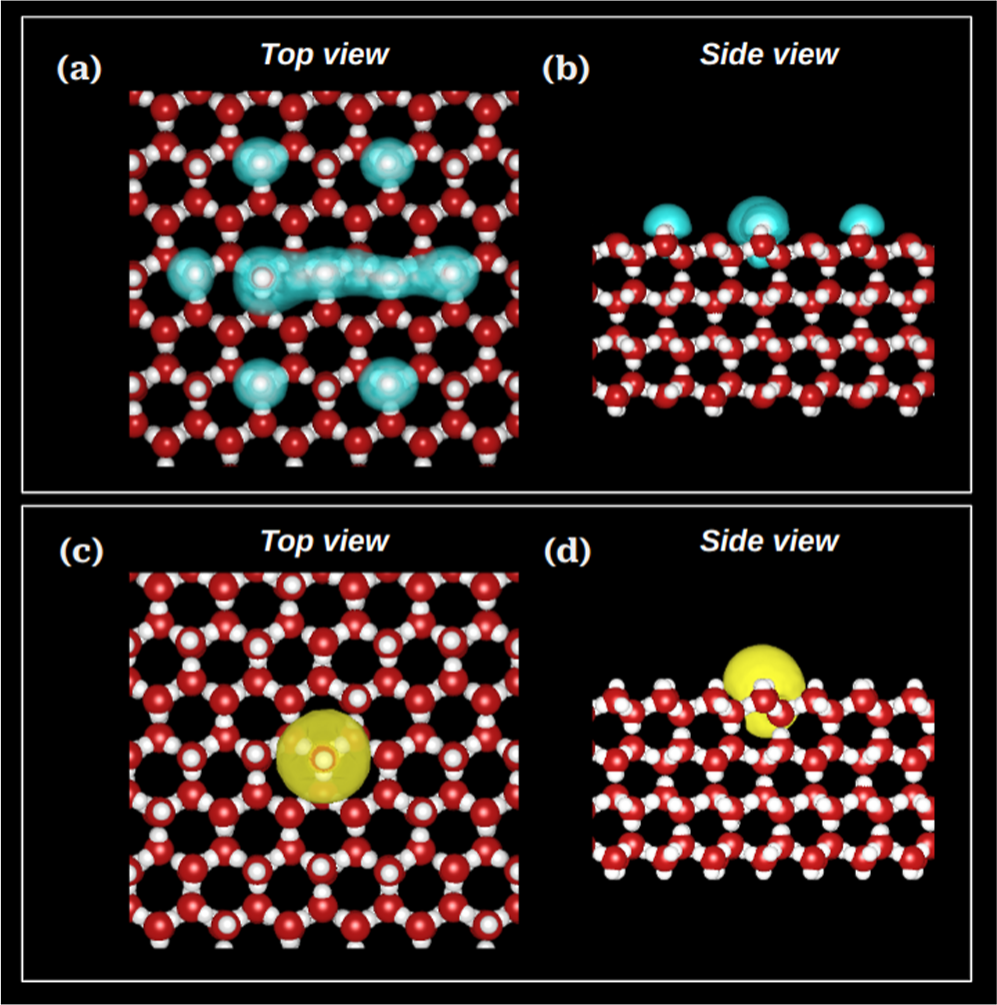
Top (a,c) and side (b,d) views of the excitonic wave functions associated with representative excitons for the Ih-striped (a,b, cyan) and Ih-low-ordered (c,d, yellow) thin-films.

The first exciton binding energy of the Ih-striped surface is about 2.9 eV, very similar to that of the Ih-low-ordered surface (3.0 eV). Interestingly, the calculated exciton binding energies are larger than those of hexagonal, cubic, and amorphous ice bulk values, and liquid water.^28,38,44,45^ This suggests that the reduced dimensionality of the thin ice layers decreases the screening and hence increases quantum effects, as expected.

## Summary and Conclusions

In this work, we investigated six models of hexagonal ice (Ih) thin films, each characterized by a distinct proton ordering quantified through the order parameter (C_OH_). Equilibrium geometries were optimized at the DFT level, and the optical properties were analyzed via reflectance anisotropy spectroscopy. The resulting spectra revealed marked differences among the models, demonstrating that subtle variations in surface hydrogen configurations can be detected through polarization-resolved optical measurements. Focusing on the two most stable surfaces, both with order parameter C_OH_ = 2.00, we performed advanced many-body perturbation theory calculations to explore their electronic and excitonic properties. GW corrections to the DFT band energies yielded a surface electronic gap of approximately 8.6 eV, in good agreement with previous theoretical studies. The present many-body analysis, although limited to two representative configurations, provides a proof-of-principle demonstration of the sensitivity of excitonic properties to proton ordering. A systematic exploration across the full range of C_OH_ values, while computationally demanding, would be required to quantitatively map the evolution of excitonic anisotropy with proton disorder.The inclusion of excitonic effects via the BSE revealed significant differences in the nature and spatial distribution of the excitonic wave functions. Notably, the optical response of the two surfaces is strongly polarization-dependent. The Ih-striped surface exhibits pronounced optical anisotropy and highly directional excitonic states, consistent with its ordered hydrogen arrangement. In contrast, the Ih-low-ordered surface shows a more isotropic excitonic behavior, reflecting its proton configuration. These differences establish optical anisotropy, and in particular RAS experiments, as sensitive probes of surface proton ordering. Moreover, advanced experimental techniques such as time-resolved ARPES,^46^ which gives the distribution of the excitons in k-spac or near-field photoluminescence optical imaging, giving the exciton wave function in real-space,^47^ could definitively shed light on the details of the proton order in ice surfaces. Our findings provide a robust theoretical framework for interpreting polarization-resolved spectroscopies of ice surfaces. By linking microscopic proton configurations to macroscopic optical observables, this work opens new avenues for experimentally distinguishing competing structural models of ice under cryogenic conditions. These insights are relevant not only for fundamental surface science, but also for applications in astrochemistry, climate modeling, and the physics of hydrogen-bonded systems.

## Methods

The equilibrium geometries of hexagonal ice thin films were optimized using DFT, as implemented in the Quantum *ESPRESSO* suite.^48–50^ The exchange–correlation functional was treated within the Generalized Gradient Approximation, using the Perdew–Burke–Ernzerhof (PBE) formulation.^51^ Long-range dispersion interactions were included via Grimme’s DFT-D2 van der Waals correction.^52^ Electronic wave functions were expanded in a plane-wave basis set with a kinetic energy cutoff of 70 Ry. Norm-conserving Martins-Trouiller PBE pseudopotentials were used. Geometry optimizations were performed until the total energy converged within 10^–5^ Ry and atomic forces were below 10^–4^ Ry/Bohr. To prevent spurious interactions between periodic slabs, a vacuum region of 12 Å was introduced along the *z*-axis. The Brillouin zone was sampled using an 8 × 6 × 1 Monkhorst–Pack *k*-point mesh,^53^ which provides well converged results (see Figure SI2 in the Supporting Information).

RAS was employed to investigate how variations in surface proton ordering affect the optical properties of hexagonal ice. The RAS spectrum was computed as^39,40^

4 $$RAS_{y-x}=\frac{R_{y}-R_{x}}{R_{0}}$$

where *R*_*i*_ is the surface reflectivity for light polarized along direction *i* and *R*_0_ is the bulk reflectivity. For bulk ice Ih, optical properties were calculated using a hexagonal supercell containing 12 atoms and a 10 × 10 × 5 Monkhorst–Pack mesh (see Figure SI1).

The optimized in-plane lattice constant for the six surface models is *a* = 4.43 Å, while the bulk ice Ih lattice parameters were *a* = 4.49 Å and *c* = 7.16 Å. These values are in good agreement with previous theoretical studies using the PBE functional^36,54^ and with experimental data,^55–57^ which typically report *a* ≈4.50 Å and *c* ≈7.35 Å. The optimized intramolecular O–H bond length was 0.99 Å for both surface and bulk models, while the intermolecular O–O distance within the hydrogen-bonded network was 2.75 Å for the surface and 2.73 Å for the bulk, in agreement with literature values.^57^ Nuclear quantum effects (NQEs), which are known to play a significant role in hydrogen-bonded systems (see for example^58,59^ ), are not explicitly included in the present calculations. Proton delocalization and zero-point motion, typically accounted for through path-integral approaches such as path-integral molecular dynamics (PIMD), can influence both the stability and the distribution of proton configurations in ice.^60,61^ In particular, NQEs are expected to enhance proton fluctuations and broaden the distribution of local hydrogen-bond environments, potentially reducing long-range proton order at the surface. As a consequence, the optical anisotropy discussed here may be partially averaged out under realistic conditions. Nevertheless, the qualitative distinction between ordered and disordered proton arrangements, and their associated optical fingerprints, is expected to remain robust. A fully consistent treatment including NQEs, for instance within a path-integral framework combined with many-body perturbation theory, represents an important direction for future work.

Many-body effects were considered only for the reduced Ih-striped and Ih-low-ordered surface models (C_OH_ = 2.00), consisting of a 4-layer hexagonal supercell with 96 atoms and a 4-layer orthorhombic supercell with 192 atoms, respectively. The DFT parameters remained unchanged, except for the Brillouin zone sampling, which used a 6 × 6 × 1 mesh for the Ih-striped model and a 2 × 4 × 1 mesh for the Ih-low-ordered model. Quasi-particle corrections to the energy gap (Δ*E*) were computed within the G_o_W_o_ approximation using the CHISIG plane-wave excited state code,^62^ interfaced with Quantum ESPRESSO. For both models, 1000 bands were included. The exchange self-energy (Σ_*X*_) was evaluated using 100,000 plane waves for the Ih-striped model and 175,000 for the Ih-low-ordered model (see Figure SI 6 in the SI for the convergence test of Σ_*X*_ with respect to the number of plane waves for the Ih-striped supercell, computed using 1000 bands and a 6 × 6 × 1 *k*-point grid). The correlation self-energy (Σ_*C*_)­and the screened Coulomb interaction *W* were computed within the plasmon-pole approximation,^63^ using 40,000 plane waves and 1000 bands for both thin films (see Figure SI 7 in the SI for the convergence test of Σ_*C*_ for the Ih-striped supercell, computed using a 6 × 6 × 1 *k*-point grid). A 2D Coulomb cutoff was applied to eliminate interactions between periodic replicas.

The G_o_W_o_ eigenvalue correction to a given DFT state was calculated using

5 $$\epsilon_{n,k}^{G_{0}W_{0}}=\epsilon_{n,k}^{DFT}+\frac{\langle n,k|\Sigma_{X}+\Sigma_{C}-V_{xc}|n,k\rangle }{1-\langle n,k|\frac{d\Sigma_{C}}{d\epsilon }|n,k\rangle }$$

Here, *V*_xc_ is the exchange–correlation potential, while the correlation self-energy Σ_*C*_ and its derivative with respect to energy are evaluated at ϵ_*n*,**k**_^DFT^. These corrections were applied at high-symmetry points and used in subsequent BSE calculations, performed with the EXC code,^64^ to obtain excitonic properties and polarization-resolved optical spectra. The BSE calculations included 45 occupied and 45 unoccupied bands for the Ih-striped model, and 60 occupied and 80 unoccupied bands for the Ih-low-ordered model.

Optical absorbance spectra were derived from the imaginary parts of the dielectric tensor components, ε_2_^*x*^(ω) and ε_2_^*y*^(ω), corresponding to light polarized along the *x* and *y* directions. The absorbance was computed using

6 $$A\left(\omega \right)=\left[\frac{\varepsilon_{2}^{x}\left(\omega \right)+\varepsilon_{2}^{y}\left(\omega \right)}{2}\right]\frac{a_{3}\cdot \omega }{c}$$

where ω is the photon energy, *a*_3_ is the effective slab thickness along the *z*-axis (in atomic units), and *c* is the speed of light. This formulation accounts for the slab geometry and polarization dependence, yielding physically meaningful absorbance spectra.

## Supplementary Material



## Notes

Includes vacuum and *k*-point convergence tests for the DFT-calculated RAS spectra performed on the ice Ih low-ordered surface (*C*_OH_ = 2.00), a schematic of the bulk supercell used as a reference for the RAS spectra calculations, the electronic band structures and projected density of states (PDOS), as well as a section on the convergence of the exchange and correlation self-energies for the Ih-striped surface (Figures SI 6 and SI 7) and the analysis of the optical transitions involved in the spectra of the Ih-striped and low-ordered Ih thin layers; To complement the qualitative analysis of the excitonic wave functions, we introduce a simple quantitative descriptor of excitonic anisotropy based on the spatial distribution of the excitonic states (PDF)
