# Supplementary material for: Optical Anisotropy as a Probe of Proton Ordering at Ice Surfaces
Source: J Chem Theory Comput. 2026 May 18;22(11):5789–96. doi: 10.1021/acs.jctc.6c00630 (PMC13255246; doi:10.1021/acs.jctc.6c00630)
Supplement: Supplementary file 1 [file ct6c00630_si_001.pdf]

# Supporting Information
## *Optical Anisotropy as a Probe of Proton Ordering at Ice Surfaces*

Alessia Muroni[1], Ding Pan[2], Marco Govoni[3], Ihor Kupchak[1,4], and Olivia Pulci[1]

[1]Department of Physics, University of Rome 'Tor Vergata' and INFN, Via della Ricerca Scientifica 1, 00133 Rome, Italy
[2]Department of Physics and Department of Chemistry, Hong Kong University of Science and Technology, Clear Water Bay, Kowloon, Hong Kong
[3]Department of Physics, Computer Science and Mathematics, University of Modena and Reggio Emilia, Via Campi 213/a, 41125 Modena, Italy
[4]V. Lashkaryov Institute of Semiconductor Physics of National Academy of Sciences of Ukraine, pr. Nauky 45, 03680 Kyiv, Ukraine

## Sec. SI 1 Vacuum Convergence Test

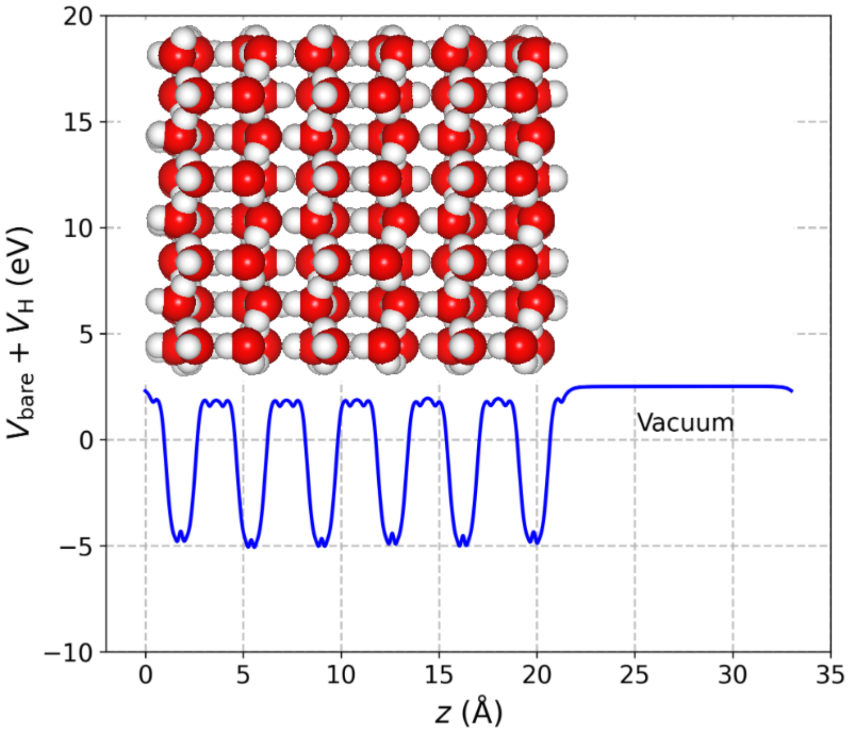


Figure SI 1: Profile of the bare plus Hartree potential ($V_{\mathrm{bare}} + V_{\mathrm{H}}$) along the $z$-direction for the ice Ih-low-ordered surface ($C_{\mathrm{OH}} = 2.00$). The 12 Å vacuum region forms a plateau, indicating negligible interactions between periodic replicas.

## Sec. SI 2 K-point convergence test for DFT RAS spectra

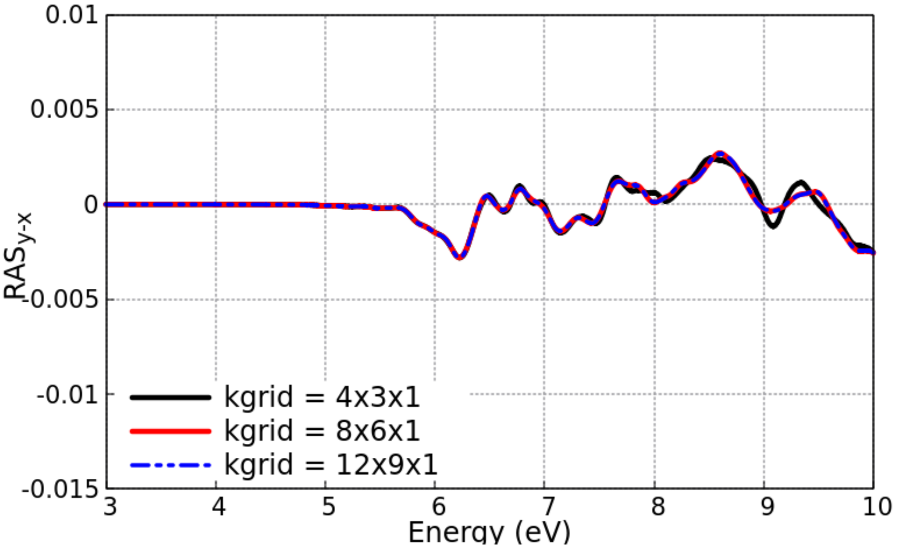


Figure SI 2: Convergence of the RAS spectra with respect to the k-point sampling for the Ih-low-ordered surface model ($C_{OH} = 2.00$).

## Sec. SI 3 Bulk Model

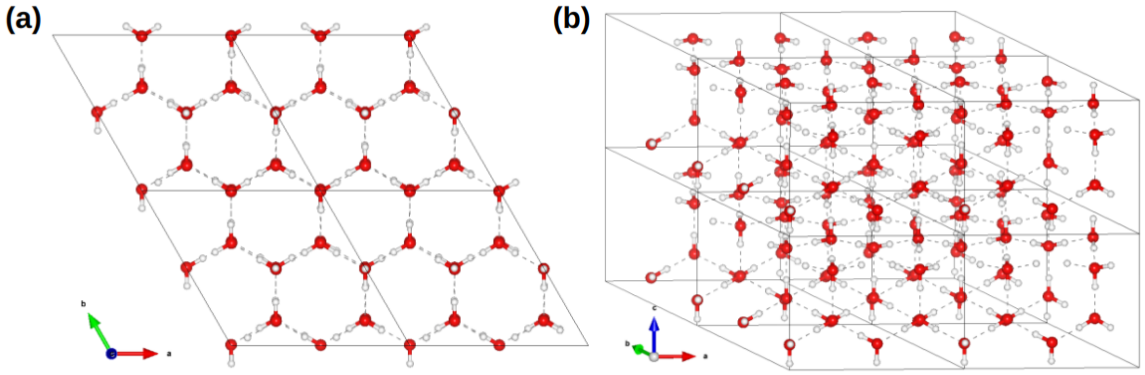


Figure SI 3: Top (a) and perspective (b) views of the Ih bulk ice supercell. Red and white spheres represent oxygen and hydrogen atoms, respectively.

## Sec. SI 4 Electronic properties of the Ih-striped and Ih-low-ordered surfaces

After optimizing the structures and obtaining their equilibrium geometries, the electronic band structures were calculated within the DFT framework along the high-symmetry paths of the respective supercells: a hexagonal path (Γ–K–M–Γ) for the *Ih*-striped surface and an orthorhombic path (Γ–X–M–X′–Γ) for the *Ih*-low-ordered surface. The resulting bands are projected onto selected atomic wavefunctions, distinguishing contributions from surface hydrogens, bulk hydrogens, and oxygens, as depicted in Figure SI 4.

The calculated band gaps for the Ih-striped and Ih-low-ordered thin-films are 5.08 eV and 5.06

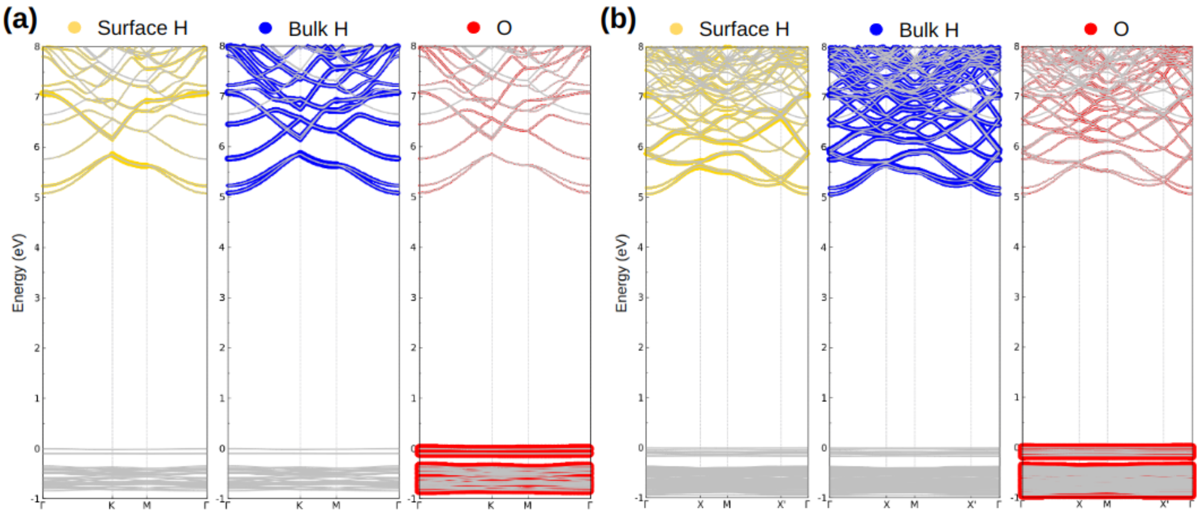


Figure SI 4: Projected electronic band structures for the Ih-striped (a) and Ih-low-ordered (b) thin-films calculated at DFT level. The projections highlight contributions from surface hydrogens (Surface H) in yellow, bulk hydrogens (Bulk H) in blue, and oxygens (O) in red, with point sizes proportional to the spectral weight.

eV, respectively. The projected band structures reveal that the valence bands are nearly flat and predominantly composed of oxygen-derived states, whereas the conduction bands are mainly associated with hydrogen-related states. These trends are further confirmed by the Projected Density of States (PDOS) of the two systems, shown in Figure SI 5.

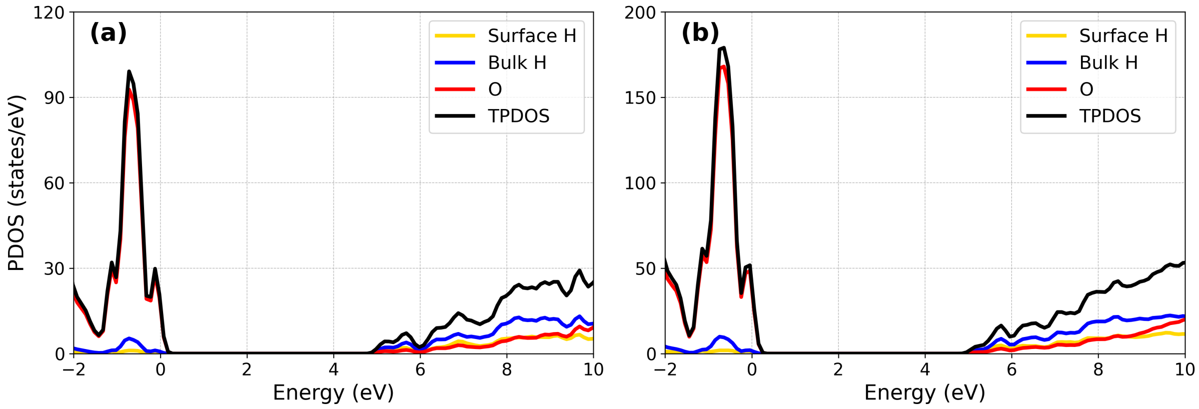


Figure SI 5: PDOS for the Ih-striped (a) and Ih-low-ordered (b) thin-films. Projections highlight contributions from surface hydrogens (Surface H) in yellow, bulk hydrogens (Bulk H) in blue, and oxygens (O) in red, along with the total density of states (TPDOS) in black.

## Sec. SI 5 Convergence Tests of Exchange and Correlation Self-Energies

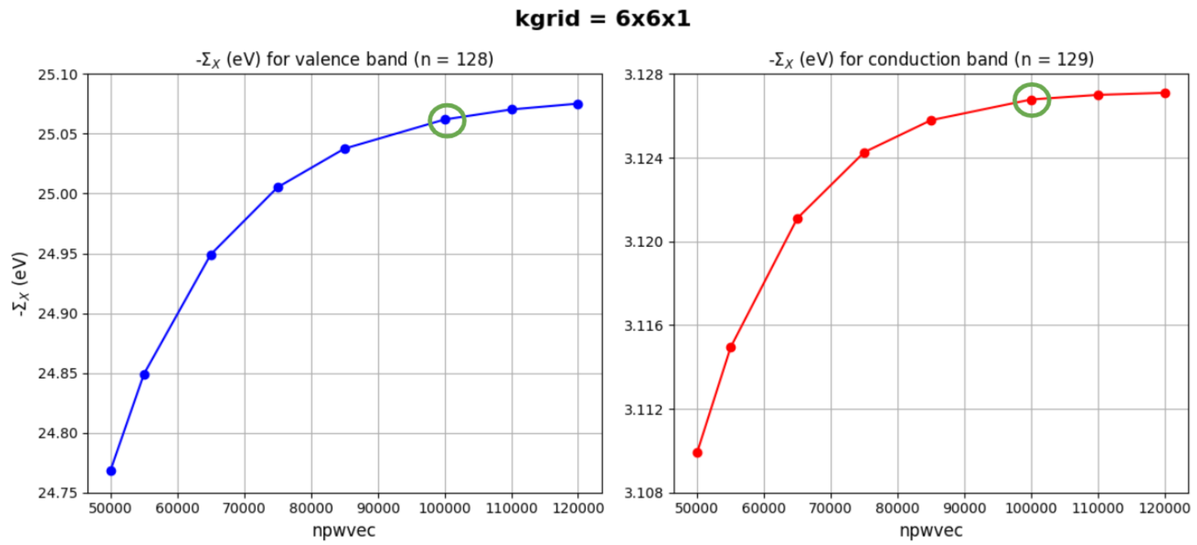


Figure SI 6: Convergence of the exchange self-energy $-\Sigma_X$ as a function of the number of plane waves (npwvec) for the Ih-striped supercell. The left panel shows the convergence for the valence band ($n = 128$), while the right panel refers to the conduction band ($n = 129$).

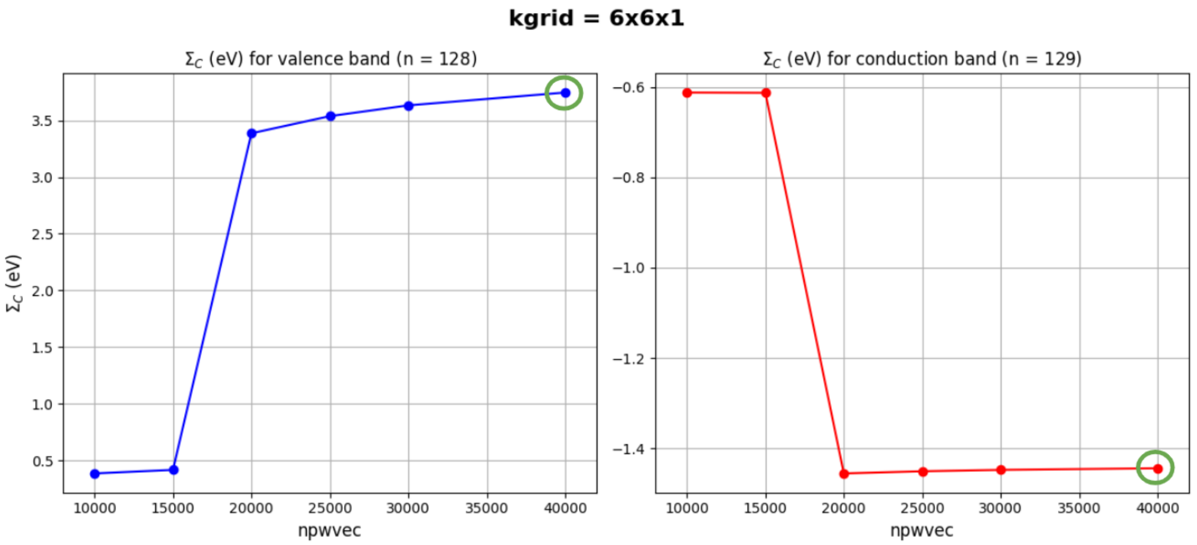


Figure SI 7: Convergence of the correlation self-energy $\Sigma_C$ as a function of the number of plane waves ($N_{\text{pw}}$) for the Ih-striped supercell. The left panel shows the convergence for the valence band ($n = 128$), while the right panel refers to the conduction band ($n = 129$).

# Sec. SI 6 Optical Properties of the Ih-striped and Ih-low-ordered surfaces

The imaginary part of the in-plane half-slab polarizability, $\mathrm{Im}[\alpha_{\parallel}^{\mathrm{hs}}(\omega)]$, was obtained from BSE calculations performed starting from GW-corrected energies. This quantity was calculated from the in-plane components of the dielectric function in the slab geometry using the relation:

$$4\pi d \cdot \mathrm{Im}[\alpha_{\parallel}^{\mathrm{hs}}(\omega)] = \frac{a_z}{2} \cdot \left( \frac{\varepsilon_{2,xx}(\omega) + \varepsilon_{2,yy}(\omega)}{2} \right) \tag{1}$$

where $d$ is the effective half-slab thickness and $a_z$ is the supercell length along the out-of-plane direction. Additionally, the in-plane optical anisotropy was analyzed through the difference between the imaginary parts of the half-slab polarizability along the $y$ and $x$ directions:

$$4\pi d \cdot \left( \mathrm{Im}[\alpha_{yy}^{\mathrm{hs}}(\omega)] - \mathrm{Im}[\alpha_{xx}^{\mathrm{hs}}(\omega)] \right) = \frac{a_z}{2} \cdot (\varepsilon_{2,yy}(\omega) - \varepsilon_{2,xx}(\omega)) \tag{2}$$

The in-plane polarizability for the Ih-striped and Ih-low-ordered surfaces, calculated at the Independent Particle Approximation (IPA), $G_0W_0$, and BSE levels, is shown in Figures SI 8(a) and SI 8(b), applying a Gaussian broadening of 0.2 eV to all spectra. Figure SI 8(c), on the other hand, reports the corresponding anisotropy along the $y$ and $x$ directions at the BSE level for both surfaces.

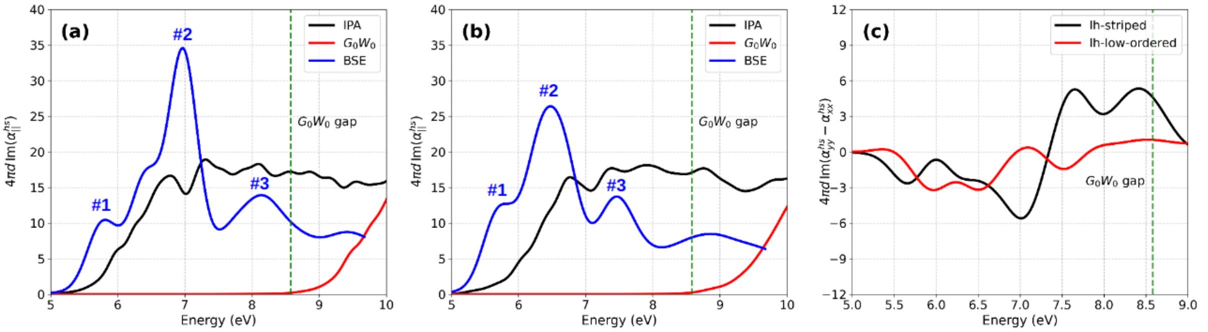


Figure SI 8: Imaginary part of the in-plane half-slab polarizability, multiplied by the half-slab thickness $d$ in atomic units, for the Ih-striped (a) and Ih-low-ordered (b) thin layers, respectively. Black line: independent particle approximation (IPA); red: independent quasi-particle spectra. Blue line: optical spectra with the inclusion of the excitonic effects, calculated within the Bethe-Salpeter Equation. Panel (c) shows the anisotropy in the imaginary part of the half-slab polarizability along the $y$ and $x$ directions, multiplied by $d$, for both surfaces at the BSE level. The vertical dashed green line, present in all panels, indicates the $G_0W_0$ band gap. All spectra include a Gaussian broadening of 0.2 eV.

A detailed analysis of the BSE peaks can offer further insight into the nature of the electronic transitions. The peaks identified in Figure SI 8 are summarized in Table SI 1. For each peak, the table reports the associated energy range and the average composition of the final states in terms of surface hydrogens (H*), which refer to hydrogens located on the analyzed surfaces, relative to all hydrogens in the slab (H). All transitions contributing to these peaks are of O $\rightarrow$ H type, so the percentage of H* effectively quantifies the surface localization of the excited electron density in the final states.

**Ih-striped**

| Peak # | Energy range (eV) | Final states (H*/H) |
|---|---|---|
| 1 | 5.7-6.1 | 15.0% |
| 2 | 6.9-7.0 | 12.9% |
| 3 | 8.1-8.2 | 15.4% |

**Ih-low-ordered**

| Peak # | Energy range (eV) | Final states (H*/H) |
|---|---|---|
| 1 | 5.7-5.8 | 21.2% |
| 2 | 6.2-6.8 | 17.1% |
| 3 | 7.2-7.5 | 17.8% |

Table SI 1: Summary of the excitonic peaks identified in Figure SI 8 for the Ih-striped and Ih-low-ordered surfaces. For each peak, the table reports the corresponding energy range and the average composition of the final states in terms of surface hydrogens (H*) relative to all hydrogens in the slab.

We quantify the anisotropy of the excitonic wavefunction by introducing a simple spatial descriptor based on the exciton electron–hole distribution. In particular, we define the anisotropy parameter as:

$$\eta = \frac{r^x_{\text{exc}} - r^y_{\text{exc}}}{r^x_{\text{exc}} + r^y_{\text{exc}}} \tag{3}$$

where $r^x_{\text{exc}}$ and $r^y_{\text{exc}}$ represent the spatial extents of the excitonic wavefunction along the x and y directions, respectively, evaluated from the exciton real-space distribution. This quantity provides a measure of the directional localization of the exciton. For the two surface models considered in this work, we obtain $\eta = 0.65$ for the Ih-striped surface and $\eta = 0.00$ for the Ih-low-ordered surface, confirming the strongly anisotropic character of the exciton in the ordered configuration and its nearly isotropic nature in the disordered case.
